# Fully automated bladder tumor segmentation from T2 MRI images using 3D U-Net algorithm

**DOI:** 10.3389/fonc.2023.1096136

**Published:** 2023-03-09

**Authors:** Diana Mihaela Coroamă, Laura Dioșan, Teodora Telecan, Iulia Andras, Nicolae Crișan, Paul Medan, Anca Andreica, Cosmin Caraiani, Andrei Lebovici, Bianca Boca, Zoltán Bálint

**Affiliations:** ^1^ Faculty of Mathematics and Computer Science, Babeș-Bolyai University, Cluj-Napoca, Romania; ^2^ Department of Urology, Faculty of Medicine, Iuliu Hațieganu University of Medicine and Pharmacy, Cluj-Napoca, Romania; ^3^ Department of Urology, Municipal Clinical Hospital, Cluj-Napoca, Romania; ^4^ Department of Medical Imaging, Iuliu Hațieganu University of Medicine and Pharmacy, Cluj-Napoca, Romania; ^5^ Department of Radiology, Faculty of Medicine, Iuliu Hațieganu University of Medicine and Pharmacy, Cluj-Napoca, Romania; ^6^ Department of Radiology, Emergency Clinical County Hospital of Cluj-Napoca, Cluj-Napoca, Romania; ^7^ Department of Radiology, Faculty of Medicine, George Emil Palade University of Medicine, Pharmacy, Science and Technology of Târgu Mureș, Târgu Mureș, Romania; ^8^ Department of Biomolecular Physics, Faculty of Physics, Babeș-Bolyai University, Cluj-Napoca, Romania

**Keywords:** fully automatic 3D image segmentation, bladder cancer, T2 MRI images, convolutional neuronal network (CNN), light 3D U-Net

## Abstract

**Introduction:**

Bladder magnetic resonance imaging (MRI) has been recently integrated in the diagnosis pathway of bladder cancer. However, automatic recognition of suspicious lesions is still challenging. Thus, development of a solution for proper delimitation of the tumor and its separation from the healthy tissue is of primordial importance. As a solution to this unmet medical need, we aimed to develop an artificial intelligence-based decision support system, which automatically segments the bladder wall and the tumor as well as any suspect area from the 3D MRI images.

**Materials:**

We retrospectively assessed all patients diagnosed with bladder cancer, who underwent MRI at our department (n=33). All examinations were performed using a 1.5 Tesla MRI scanner. All images were reviewed by two radiologists, who performed manual segmentation of the bladder wall and all lesions. First, the performance of our fully automated end-to-end segmentation model based on a 3D U-Net architecture (by considering various depths of 4, 5 or 6 blocks) trained in two data augmentation scenarios (on 5 and 10 augmentation datasets per original data, respectively) was tested. Second, two learning setups were analyzed by training the segmentation algorithm with 7 and 14 MRI original volumes, respectively.

**Results:**

We obtained a Dice-based performance over 0.878 for automatic segmentation of bladder wall and tumors, as compared to manual segmentation. A larger training dataset using 10 augmentations for 7 patients could further improve the results of the U-Net-5 model (0.902 Dice coefficient at image level). This model performed best in terms of automated segmentation of bladder, as compared to U-Net-4 and U-Net-6. However, in this case increased time for learning was needed as compared to U-Net-4. We observed that an extended dataset for training led to significantly improved segmentation of the bladder wall, but not of the tumor.

**Conclusion:**

We developed an intelligent system for bladder tumors automated diagnostic, that uses a deep learning model to segment both the bladder wall and the tumor. As a conclusion, low complexity networks, with less than five-layers U-Net architecture are feasible and show good performance for automatic 3D MRI image segmentation in patients with bladder tumors.

## 1 Introduction

Bladder cancer (BCa) represents the tenth most frequent malignancy, with over 539.000 new cases being diagnosed worldwide each year (1). At diagnosis, 75% of tumors are limited to the mucosa and submucosal layer of the bladder wall (non-muscle invasive bladder tumor - NMIBC), whereas in 25% of cases the tumor is muscle-invasive (MIBC) (2). The choice of treatment depends on the accurate tumor staging, with bladder-preserving treatment being recommended in NMIBC and radical excision in MIBC. Therefore, precise assessment of the muscular layer invasion is mandatory.

Staging of BCa requires preoperative imaging and endoscopic evaluation. Transurethral resection of the bladder tumor (TURBT) confirms the depth of invasion and provides the histopathological diagnosis (3). However, due to tumoral heterogeneity and variation in resection techniques, upstaging from NMIBC to MIBC has been reported in up to 32% of cases, when compared to the cystectomy specimen (4). Although considered a minimally-invasive procedure, TURBT it is not voided of perioperative complications, such as urinary tract infections (24%), hemorrhagic events requiring blood transfusion (13%) and bladder wall perforation (5%), with an overall mortality being estimated at 1.3% (5). Moreover, the delay from the initial diagnosis to radical treatment in MIBC due to TURBT and confirmatory reTURBT can have a negative impact upon the oncologic outcomes of these patients, with potential tumor progression and metastasis during this time. As such, a non-invasive imaging method to differentiate between NMIBC and MIBC might be of significant impact.

Recently, multiparametric magnetic resonance imaging (mpMRI) of the urinary bladder has been developed, with studies reporting a sensitivity and specificity of 90% and 78% for bladder tumor staging, respectively (6). It is, however, operator dependent, as inter-reader agreement varies throughout studies and MRI acquisitions: 80.4% for T2 weighed images (T2WI), dropping to 71.4% and 55.4% for diffusion-weighed images (DWI) and dynamic contrast enhanced (DCE) scans, respectively (7).

Taking these limitations into consideration, precise delineation of the bladder wall as well as of the bladder tumor are important steps toward non-invasive BCa staging. Previous preliminary studies have anticipated the role of artificial intelligence (machine-learning and convolutional networks) in increasing the BCa detection and staging performance of mpMRI, reporting an accuracy of 87.9% (8).

**Figure 1:**
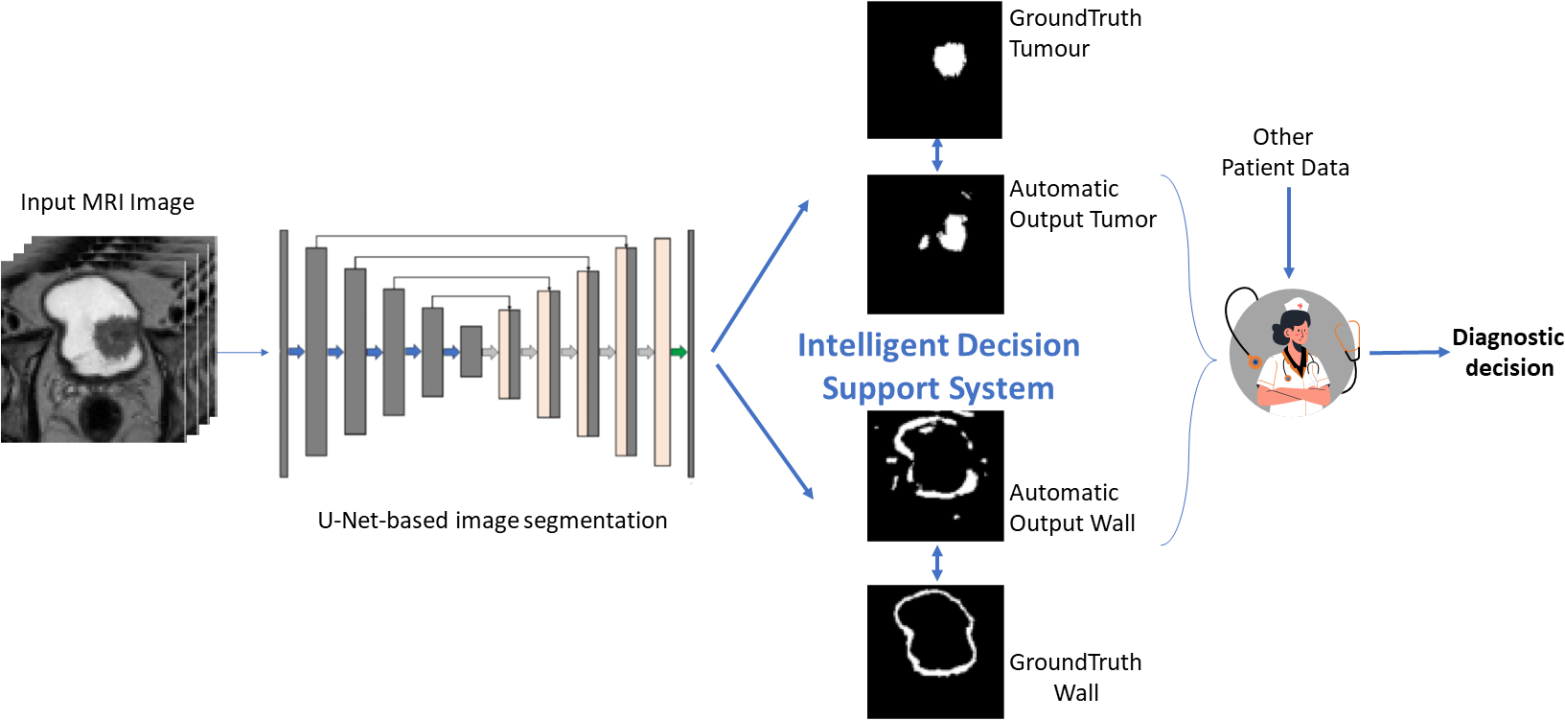
Graphical description of the proposed approach.

In medical segmentation, the most used segmentation neural network is U-Net. Its popularity stems from the fact that it produces a satisfactory segmentation with very few data samples available. As a result, it has served as the foundation for numerous medical segmentation models. The first architecture that we investigate was proposed in Dolz et al. (9), and it was developed specifically for the task of bladder segmentation. Throughout this paper we will refer to this model as the U-Net-5 (a U-Net with 5 blocks) for comprehension reasons. The U-Net-5 can be considered the state-of-the-art network for bladder segmentation since it can segment simultaneously both bladder walls and tumors. Starting from this model, we developed a novel model that directly processes the 3D MRI input (**Figure 1**) and we investigated whether a slim network (with fewer blocks) or a deeper one (with more blocks) could perform better in the segmentation of bladder tumors.

In this context, we aimed to develop an advanced model to automatically detect the bladder wall and the tumor from the MRI images using a 3D U-Net neural network. A future step will consist of proposing a metric to quantify muscle invasiveness of the tumor based on the obtained segmentations.

## 2 Materials and methods

### 2.1 Patient population

This observational study was conducted in accordance with the Declaration of Helsinki (as revised in 2013) and obtained ethical approval from the local Ethics Committee (Nr. 26/2020). Individual consent for this retrospective analysis was waived. The authors are accountable for all aspects of the work in ensuring that questions related to the accuracy or integrity of any part of the work are appropriately investigated and resolved.

### 2.2 Clinical assessment

#### 2.2.1 MRI

The patients with *de novo* Bladder Cancer were referred to MRI evaluation. All MRI examinations were performed before the endoscopic resection of the bladder tumor. The examinations were performed in a single institution, using a 1.5 Tesla MRI scanner (General Electric Optima 360MR Advance System). The patients were asked to void 1-2 h before the examination and then drink 500 mL of water 30 min before the examination for optimal bladder distention. The protocol included: three T2 weighted turbo spin-echo (TSE) sequences in the sagittal, coronal, and oblique-axial high-resolution planes, a T1 TSE weighted sequence in the axial plane. Diffusion-weighted images were obtained in axial planes using EPI sequences at b values of 50, 400, 800, 1000, 1500 and the image software automatically calculated apparent diffusion coefficient maps (ADC). An unenhanced axial T1 VIBE sequence acquisition was performed and was followed by additional axial T1 VIBE scans after contrast administration. The contrast agent gadobutrol (Gadovist^®^ 1.0; Bayer Schering Pharma AG, Berlin, Germany) was administered employing the free-hand technique, using a dose of 0.1 mmol/kg^−1^. Dynamic contrast enhanced axial 3D T1WI were immediately acquired after contrast administration.

All oblique-axial high-resolution T2-WI TSE image were retrieved from a picture archiving and communication system (Pixeldata PACS, Romania) for image segmentation. Two radiologists: one radiology resident and a senior radiologist with 10 years of experience in urogenital MRI reviewed all images and reached a consensus about the tumor location. Afterwards, the radiology resident segmented the whole tumor volume, by manually delineating the lesion on each consecutive slide. The segmentations were then independently reviewed by the senior radiologist and adjustments were made when necessary. Both radiologists were blinded to the pathological results of biopsy specimens. The segmentation of the tumors was performed using a designated, open-source software, 3D Slicer, version 4.11.2 (available at https://www.slicer.org/).

All patients underwent TURBT in the same institution using bipolar/monopolar complete resection of all bladder tumors. ReTURBT was performed according to the indications of the current European Association of Urology Guidelines. The retrieved specimens from TURBT were immersed in 4% formaldehyde fixation solution and kept at 4°C overnight. After paraffin embedding, hematoxylin - eosin stain was performed, followed by immunohistochemical stains, as needed. Pathology results have been assessed by the same two pathologists with extensive experience in urological disease.

### 2.3 Software development

#### 2.3.1 Data preparation

The specialist provided segmentation image dataset of each patient has been divided into 2 files, one designated for the bladder mask wall, and one for the tumor mask. If multiple tumors were detected in the same patients, the largest one was considered further as the index tumor. The division was obtained using the 3DSlicer application, which converted the segmentation from the segmentation specific format file (.seg.nrrd) into 2 files in the NRRD (.nrrd) format. Each file was transformed into a 3D Numpy array, whose slices are the individual images. From this point on, when referring to a dataset we use the term 3D Numpy array. The arrays go through a series of processes to be used as training data. First, the region of interest is extracted by using both masks of the bladder together (wall and tumor) as well as an auxiliary mask formed by combining the two. From the data we manually extracted a ROI of 300 x 300 x 32. We observed from looking at the data that the ROI of each one was found in the top-center, more exactly from 100 to 400 pixels in width and length. Another possibility for our data could have been to crop the ROI based on the segmented ground-truth image, but such an approach was not feasible for the testing stage when new volumes are provided by the scanner (without being annotated by the specialist).

#### 2.3.2 Data augmentation

The size of the entire cohort of original MRI volumes was 33. Some of them were used for training the segmentation algorithm, while others for testing it. Before performing the training process, we chose to use different methods for data multiplication: rotation of the arrays by a certain degree taken at random, flipping the arrays and elastic transformations. These augmentation operations are applied to the data containing the original images as well as to the data containing the bladder wall masks and the tumor masks, respectively. For each one of the training patient’s volumes, ***na*** new volumes were added to the training data and 1 to the validation data. Individual data for the two augmentation scenarios (***na*** = 10 and ***na*** = 5) is presented in **Table 1**.

**Table 1:** Data augmentation summary (number of augmented datasets used for training).

Augmentation parameter	Training setup with x original volumes
***na*** = 10	x + x * 10
***na*** = 5	x + x * 5

**Figure 2:**
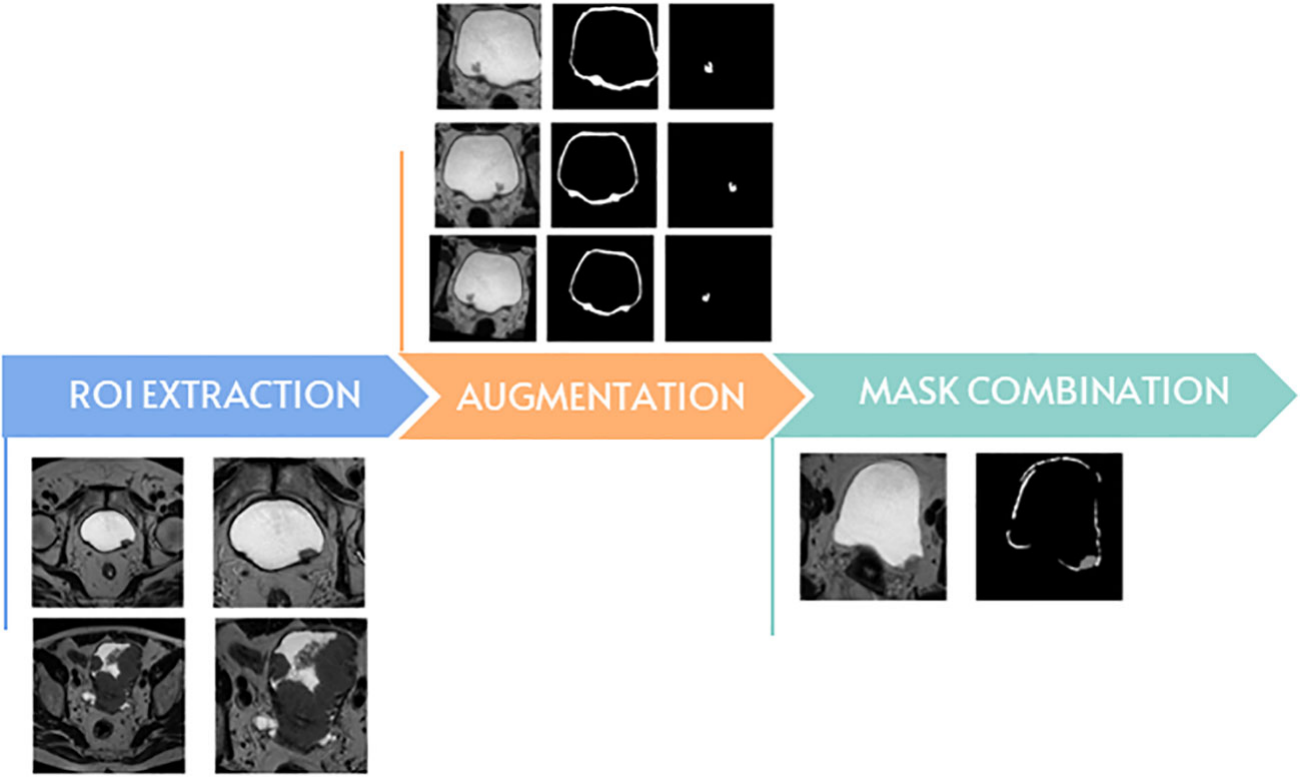
The main steps of data preparation.

In the rotation method, 3 values are chosen at random between 5 to 10 for the positive angles and 3 numbers between -10 to -5 for the negative angles. The arrays are rotated with the randomly chosen angles and added to the data, considering that 2 out of the 4 positively rotated arrays, and 3 out of the 6 negatively rotated arrays are flipped horizontally. Two elastic transformations were applied to each image dataset and their flipped variant which resulted in four new datasets. Due to issues such as limited RAM, we made the choice to reshape the data from 300 x 300 x 32 to 128 x 128 x 32. The last step in the preprocessing part was to normalize the data by making the mean close to 0 and the standard deviation close to 1. In the arrays containing the masks the value 1 is replaced with the value 255 for the wall, and 125 for the tumor. We assembled the two masks in a unitary array so that they could be used for training (**Figure 2**).

#### 2.3.3 Model creation

**Figure 3:**
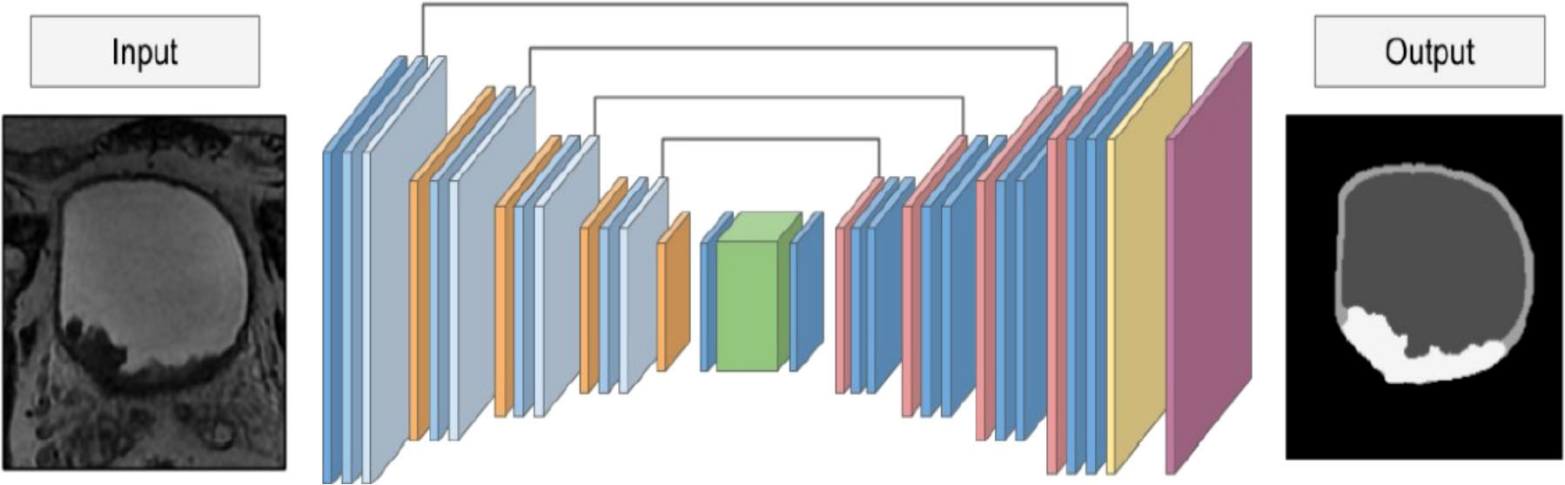
The architecture of the U-Net-5 model.

In medical segmentation, the most used neural network is the U-Net, since it produces a satisfactory segmentation with very few data samples available (**Figure 3**). The model we created was inspired by the state-of-the-art architecture proposed in the article by Dolz et al. (9). The model consists of two main parts: Encoder and Decoder. Unless explicitly specified, each convolutional layer is followed by BatchNormalization and activated with the PReLU function. The encoder is composed as follows:

- 15 convolutional layers in which the dilation rate alternates from 1 to 2 and 4 respectively for each level as it descends.
- The first layer of each depth level has the strides equal to 2, in other words the filter will move 2 pixels at a time through the volume which will halve the volume.
- A “bridge” block consisting of 2 convolutional layers and a residual block.
- the residual block is in turn composed of 2 convolutional layers, they have a dilation rate of 1 and are not followed by normalization, only activated by PReLU.

The decoder is composed as follows:

- An UpSampling3D layer is applied at the beginning of each depth, to return to the initial shape of the volume input in the model.
- After applying the UpSampling3D, the current layer is merged with its respective counterpart (the layer with the dilation rate equal to 4) from the Encoder portion of the neural network followed by 2 convolutional layers.
- Finally apply a convolutional layer with 3 filters in which the kernel size is (1,1,1) (there are 3 filters because the number of classes in which we want to divide the image is 3) with BatchNormalization and PReLU followed by a Softmax activation layer.

As done by Dolz et al. (9), Adam was used as an optimizer with a learning rate of 5e^-4^ and, when compiling, the categorically cross-entropy function was used to measure loss.

#### 2.3.4 Model evaluation

For evaluating the performance of the segmentation models, we used the Dice similarity metric or Dice similarity coefficient, which is one of the most common metrics for the purpose of calculating the overlap between two predictions for medical segmentation:


$$Dice\;=\;\frac{2\;\cdot \left|A\cap^{\;}B\right|}{\left|A\;\cup^{\;}\;B\right|}$$

where A represents the ground-truth image, B represents the predicted image, $\left|A\cap^{\;}B\right|$ counts the common segmented elements of A and B, $\left|A\cup^{\;}B\right|$ and counts all the elements of A and B.

The original testing data and its masks went through a similar process to the training data. Two post-processing stages were applied to the model’s output, which contained the probability maps for each altered version of the original MRI data. First, a bilateral filter was used to exclude all but the most intense regions, and then a threshold was set so that the values were only 1 or 0.

## 3 Results

A total of 33 patients with 33 index tumors were analyzed. Demographic data are synthetized in **Table 2**.

**Table 2:** Characteristics of the study population (Quantitative data are given as mean [range].

Variable	Number
**Age, years [range]**	65.93 [39-82]
Gender	
Male	28
Female	5
Smoker	
Yes	9
No	24
Tumor site	
Right lateral wall	11
Left lateral wall	11
Anterior wall	2
Right ureteral orifice	4
Left ureteral orifice	4
Bladder trigone	1
Invasion of muscularis	
NMIBC MIBC	27 6
pT	
pTa	15
pT1	12
pT2	6
Grade	
Low grade	15
High grade	18

Qualitative data are given as numbers, NMIBC – non-muscular invasive bladder cancer, MIBC – muscular invasive bladder cancer).

First, we tested the standard U-Net architecture (U-Net-5), as well as two alternate versions of it, one which contained an additional level (U-Net-6), and the other one with a subtracted level in comparison to the original architecture (U-Net-4).

**Figure 4:**
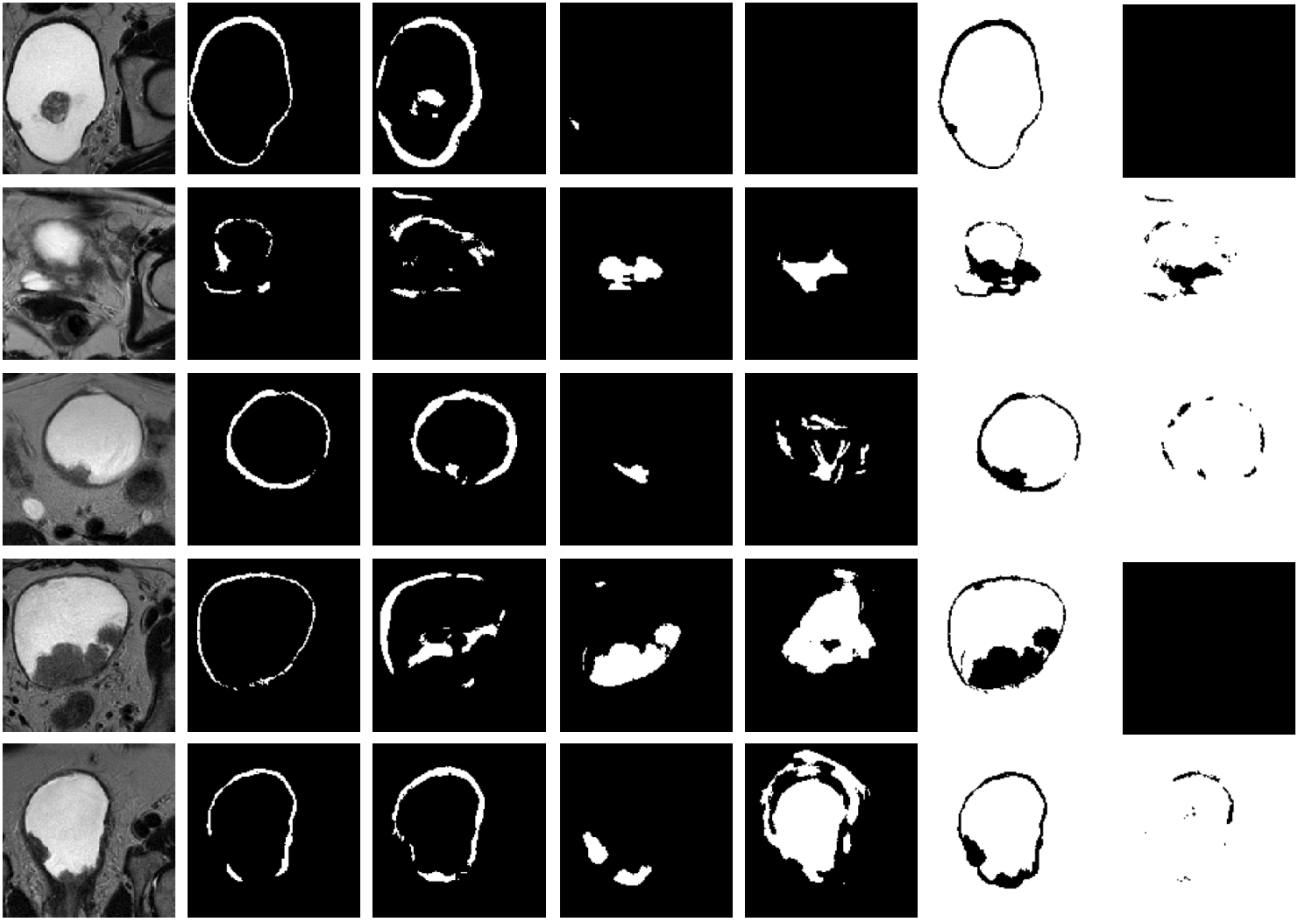
Representative results on 5 test datasets by using a model based on U-Net-5 trained on 7 original MRIs with 5 augmentations. Original slice, prostate (ground-truth and prediction), tumor (ground-truth and prediction), background (ground-truth and prediction). Each row corresponds to a patient and the columns to the original image, ground truth of the wall, predicted wall, ground-truth of the tumor, predicted tumor, ground-truth of the background and predicted background, respectively. For each patient the slice corresponding to the best tumor segmentation was plotted.

The training time differed between architectures. Each U-Net-based model was trained until the Dice coefficient for the validation dataset stopped improving, thus the best model was saved for each improved validation value. After testing, U-Net-5 reached a good overall value around 50 epochs, in around 30 minutes of training from which point on the model stopped improving. When we trained the modified versions of the U-Net, neither model reached the performance of the original one. In terms of training epochs, the model with the additional level (U-Net-6) stopped after 17 epochs, whereas the model with the decreased level (U-Net-4) finished after 33 epochs. In this convergence experiment, the training dataset was composed from datasets of 7 patients, derived by augmentation operations for ***na*** = 5 and ***na*** = 10 (e.g., **Figure 4** using U-Net-5 with 5 augmentations and **Supplementary Figures 1–5**). The average results obtained by all the trained models applied on the 5 test MRI volumes are presented in **Table 3**.

**Table 3:** Comparison of the 3 U-Net architectures, with different augmented data sample sizes.

Model	Augmentation parameter (*na*)	Overall Dice	Bladder Wall Dice	Tumor Dice
U-Net-4	10	0.884	0.783	0.878
	5	0.865	0.742	0.861
U-Net-5	10	0.902	0.836	0.879
	5	0.885	0.787	0.876
U-Net-6	10	0.902	0.835	0.880
	5	0.876	0.761	0.877

Overall, no significant difference was observed between models. From the performance and complexity point of view, the U-Net-5 model with a larger augmented training set (***na*** = 10) performed better in terms of automated segmentation, needing however an increased learning time.

**Figure 5:**
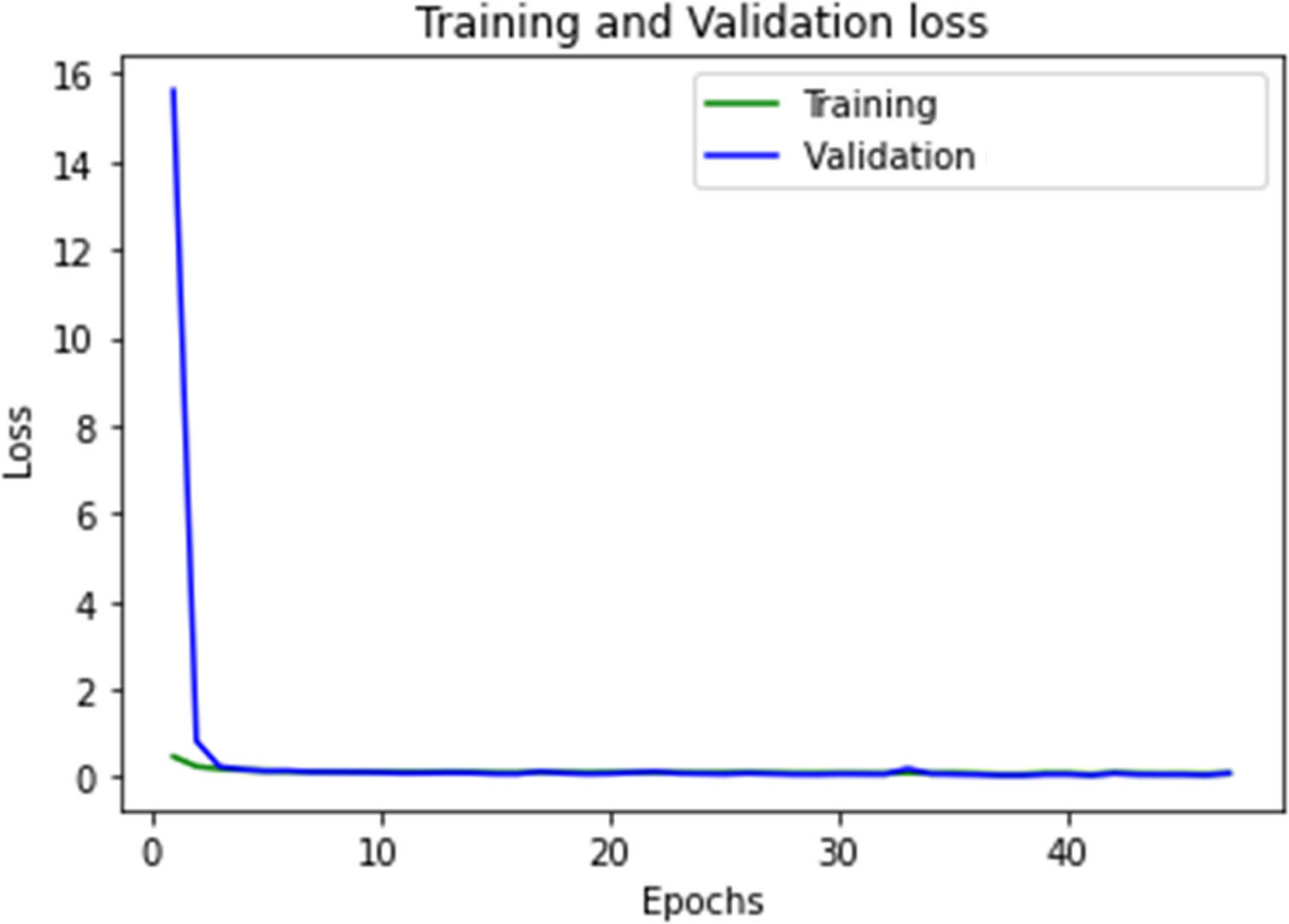
The convergence of the U-Net-5 model.

As a supplementary measure, we addressed the loss. **Figure 5** illustrates the convergence of the learning for the U-Net-5 architecture (with 7 MRI images used as training data and 5 augmentations for every original image). We can observe the improvement of the loss and its rapid convergence to 0. As the loss function measures the model’s error, it returns a value between 0 and 1, with 0 indicating that the prediction is the same as the ground truth and 1 indicating that they are radically different. In our case the loss was computed by using categorically cross-entropy function:


$$Loss=-\sum_{i=1}^{OutputSize}y_{i}\log{\hat{y}}_{i}$$

where *y_i_* represents the true value, or the ground truth, of the voxel and ${\hat{y}}_{i}$ the output of the model for the voxel in respect to the i label. In this formula a voxel has the value of 1 if it belongs to the i class and 0 otherwise. The number of classes was 3 (bladder wall, bladder tumor, inner region of the bladder).

**Figure 6:**
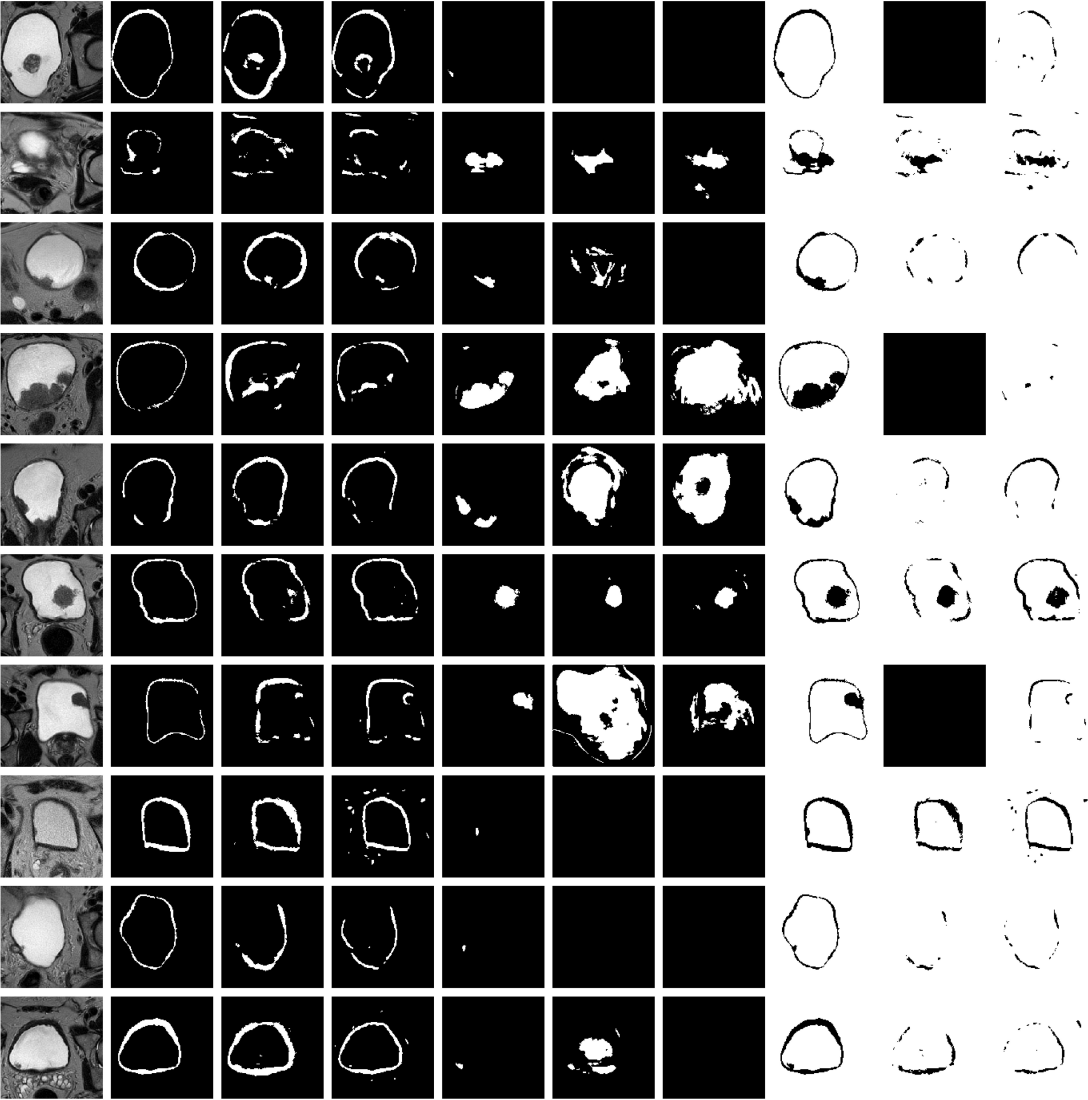
Representative results on 10 test datasets by using a model based on U-Net-5 trained on 7 original MRIs (U-Net-5-trained-7) and by using a model based on U-Net-5 trained on 14 original MRIs (U-Net-5-trained-14). Original slice, prostate (ground-truth and two predictions), tumor (ground-truth and two predictions), background (ground-truth and two predictions). Each row corresponds to a patient and the columns to the original image, ground truth of the wall, predicted wall by U-Net-5-trained-7, predicted wall by U-Net-5-trained-14, ground-truth of the tumor, predicted tumor by U-Net-5-trained-7, predicted tumor by U-Net-5-trained-14, ground-truth of the background, predicted background by U-Net-5-trained-7 and predicted background by U-Net-5-trained-14, respectively. For each patient the slice corresponding to the best tumor segmentation by U-Net-5-trained-7 was plotted. (The first column corresponds to the original slice, the second column to the ground truth of the wall mask, tumor mask and background). For each patient the slice corresponding to the best tumor segmentation obtained by the UNet-5 model trained on 7 original MRIs was plotted. Values are the dice coefficients for the plotted slice).

The second experiment focused on investigating the effect of the original training data size on the segmentation performance. To this aim, we considered the U-Net-5 model and we trained it in two different settings: one that was using 7 original volumes as the base of training data (**Figure 4**) and one that was using 14 original datasets (**Figure 6**). In both cases an augmentation process with ***na*** = 5 was performed. For U-Net-5 with 7 MRI original volumes used for learning, the training duration was roughly 30 minutes, whereas using 14 MRI original volumes for learning the duration was around 60 minutes.

In **Table 4** the segmentation performance obtained by the above-described trained models for 5 test MRI volumes are presented.

**Table 4:** Results of 5 test MRI volumes obtained by the U-Net-5 model trained in two different setups.

Training setup	Overall Dice	Bladder Wall Dice	Tumor Dice
7 original MRI volumes	0.885	0.787	0.876
14 original MRI volumes	0.903	0.838	0.881

The presented Dice values are averages over all slices of a test volume and all volumes.

We observed that an extended training dataset improves the segmentation performance for both target volumes (bladder wall and bladder tumor), as well as for the whole organ. The overall enhancement is 2% if the Dice of the entire image is considered (The enhancement is computed as the relative difference of the Dice coefficient obtained by using 14 training volumes and that obtained by using 7 training volumes). However, we can notice that the Dice coefficient for the bladder tumor increased by 1% when the training dataset was enhanced, while the same metric boosts by 6% for the bladder wall.

**Figure 7:**
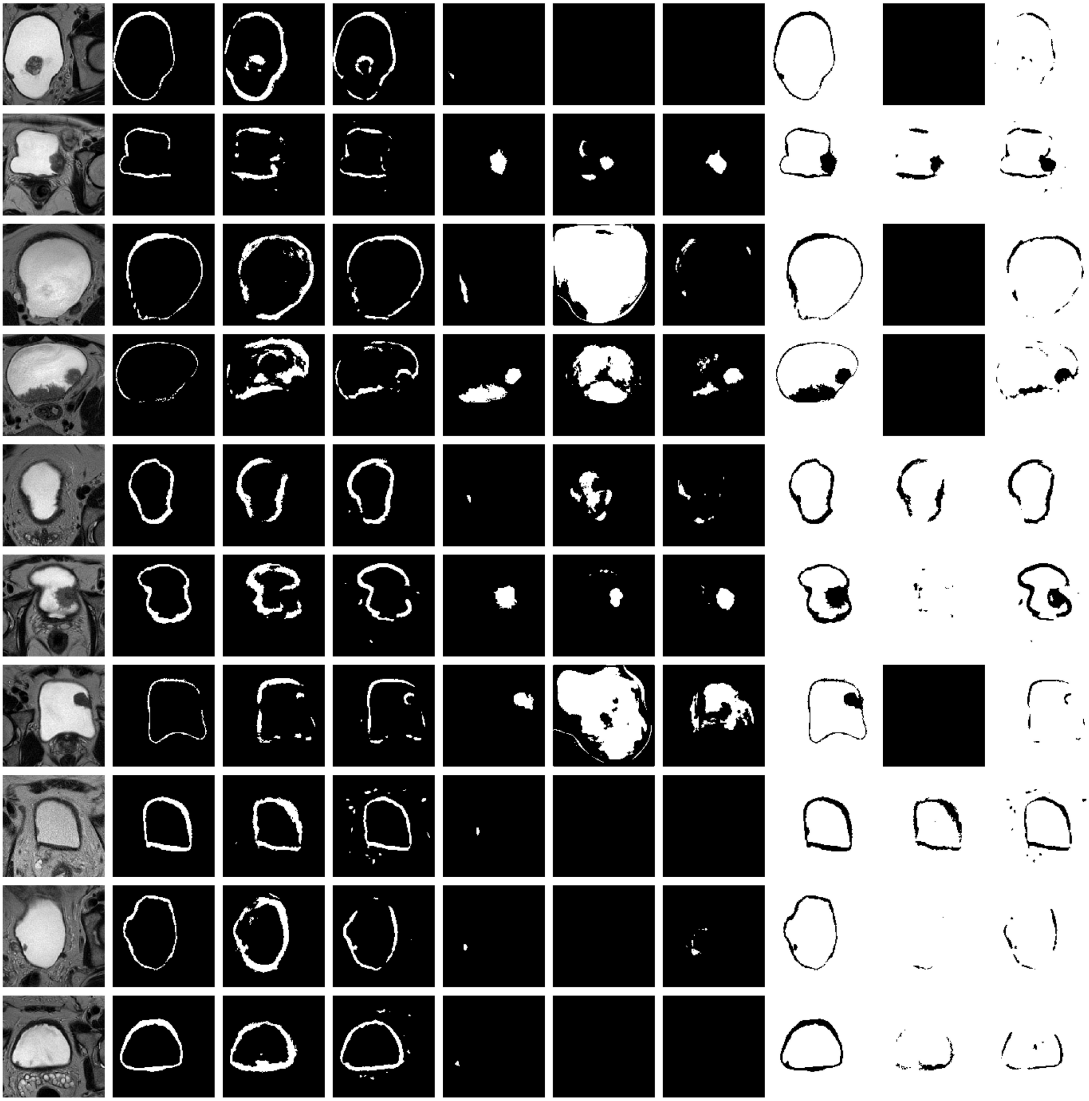
Representative results on 10 test datasets by using a model based on U-Net-5 trained on 7 original MRIs (U-Net-5-trained-7) and by using a model based on U-Net-5 trained on 14 original MRIs (U-Net-5-trained-14). Original slice, prostate (ground-truth and two predictions), tumor (ground-truth and two predictions), background (ground-truth and two predictions). Each row corresponds to a patient and the columns to the original image, ground truth of the wall, predicted wall by U-Net-5-trained-7, predicted wall by U-Net-5-trained-14, ground-truth of the tumor, predicted tumor by U-Net-5-trained-7, predicted tumor by U-Net-5-trained-14, ground-truth of the background, predicted background by U-Net-5-trained-7 and predicted background by U-Net-5-trained-14, respectively. For each patient the slice corresponding to the best tumor segmentation by U-Net-5-trained-7 was plotted.

As a next step, the previously trained segmentation models were applied to 9 new MRI volumes obtained from independent patient datasets (**Figure 7**). The average results obtained over all 14 test volumes are summarized in **Table 5**. The relative difference of the segmentation quality was increased by 4% at the entire MRI image level. Whereas, for the bladder wall we obtained an increase by 17%, but with only 1% increase at the level of the tumor.

**Table 5:** Results of the 5 + 9 test MRI volumes obtained by U-Net-5 model trained in two different setups.

Training setup	Overall Dice	Bladder Wall Dice	Tumor Dice
7 original MRI volumes	0.739	0.590	0.638
14 original MRI volumes	0.772	0.688	0.645

The presented Dice values are averages over all slices of the test volume and all volumes.

For most of the cases both, the wall and the tumor were properly localized by the algorithm, thus the precondition for quantitative analysis of the tumor localization as compared to the bladder wall is satisfied. The above presented promising results could be transformed as prerequisites for the next step of proposing a metric to quantify muscle invasiveness of the tumor based on the obtained segmentations to differentiate between non-muscle invasive and muscle-invasive bladder tumor (NMIBC vs MIBC).

## 4 Discussions

The U-Net-5 model with a large training set (***na*** = 10) performed best in terms of automated segmentation of bladder MRI images (only T2-WI TSE sequences), as compared to U-Net-4 and 6. However, increased time for learning was needed as compared to U-Net-4. An extended dataset for training leads to significantly improved segmentation of the bladder wall, but not of the tumor. Probably the improvement for tumor segmentation will be seen after an even more extensive dataset, due to high heterogeneity of its appearance.

Preoperative detection of bladder tumors and their extension into the muscle layer has the potential of guiding future therapeutic strategies in a non-invasive way. Before mpMRI, contrast-enhanced computer tomography was used for bladder tumors segmentation. Although this imaging modality reaches a good accuracy in terms of automatic tumor detection (84.2% on a study database of 182), in terms of preoperative grade assessment, the accuracy drops to 77.9% (10), this being mainly attributed to the limited contrast between adjacent soft tissue structures (11).

Previous articles based on MRI studies have obtained good, reproductible results. Xu et al. (8) developed a model comprised of 62 cancerous regions and 62 macroscopically ‘normal’ bladder wall, meant to assess the heterogeneity of the tumoral tissue, compared to the adjacent structures. The algorithm was modeled using 3D reconstructions of manually segmented regions of interest, with 29 optimal high-order features being extracted and further augmented using Synthetic Minority Over-sampling Technique (SMOTE). The reported sensitivity, specificity and accuracy were 89.67%, 87.8% and 88.74%, respectively. Similarly, Shi et al. (12) analyzed the bladder carcinoma, the bladder wall of patients diagnosed with urothelial malignancies, as well as the bladder wall of healthy patients. Apart from reaching an accuracy of 86.97% in terms of tumoral detection using a computer-assisted diagnosis tool based on machine learning, they have also concluded that the features extracted from macroscopically intact bladder walls of oncological patients vary substantially compared to the healthy subjects, being interpretated as because of vascular invasion and fibrosis.

While bladder segmentation is in the spotlight of current research, there are only a few solutions in current literature to this challange (13). The appearance of the bladder on the MRI images, its indistinct borders, and the distinct forms of tumors make the automatic segmentation of the bladder wall and tumor a challenging task. The design proposed by Dolz et al. (9) is one of the most widely used architecture with T2 weighted images as input. The authors started with the U-Net architecture and enhanced it by employing progressive dilated convolutions, which were introduced contextually for each module. The network handles multi-region bladder segmentation in which the regions are the Interior Wall (IW), Outer Wall (OW), and the tumor. Hammouda et al. (14) took a different strategy by employing DeepMedic (15) and presented two architectures, one which uses 2D images (16) and the other using 3D volumes as input (14). Using affine transformation followed by bsplines (17) on MRIs and Ground Truth, a manually generated adaptable form was created, which was then used to create the shape prior probability. The shape prior probability approximating the shape of the bladder. Both the MRIs and the shapes prior probability were used as input. These two proposed models focused on multi-region segmentation of bladder cancer structures. In another approach Hammouda et al. (16) used a 3D Convolutional Neural Network with ten cascade layers, eight of which used normalization and a 3x3x3 kernel size. For post-processing they employed a fully connected conditional random field (CRF) layer. In comparison to the other two models, it only segments the bladder’s IW and OW, leaving the tumor out. Whilst these works gave the best results, they are missing key features that would allow them to be replicated, such as the type of affine transformation, initial learning rate, type of optimizer used, batch size, image size and loss. Each model had a different number of patients for training, i.e. their models used 20, 10 and, 17 patients, respectively. Liu et al. (18) proposed a modified U-Net architecture which has dilated convolutions, lateral connections, and multi-scale predictions.

A direct comparison of the segmentation performances obtained on our datasets to other results from literature (obtained by other algorithms or by using other datasets) is difficult since the image characteristics (signal-noise ratio, noise type, the repetition and echo time, fine tuning of imaging parameters, etc.) vary substantially. Furthermore, the investigated models (U-Net-4, U-Net-5, U-Net-6) were validated on other medical images (e.g. prostate cancer). Future perspectives of radiomics in terms of bladder cancer management reside in their integration into risk assessment nomograms, aiming to predict more accurately the tumoral staging and chemotherapy response. Zheng et al. (19) integrated texture analysis features extracted from T2WI and DCE acquisitions with clinical parameters such as age, sex, number of tumors and their maximum size, as well as the attributed VI-RADS score. The nomogram was tested on 129 patients and validated on 56. The combined model reached an accuracy of 93% in terms of differentiating muscle invasion preoperatively. Finally, radiomic features have the potential of predicting neoadjuvant chemotherapy response, defined as ≤ pT1 on the pathological report from radical cystectomy (20). Based on aggressiveness patterns derived from T2WI, ADC and DWI, combined with clinical staging, the proposed nomogram reached sensitivity, specificity, and accuracy of 94.4%, 94.1% and 94.3%, respectively. However, both studies underline the need for prospective validation on larger cohorts of patients.

Recently, artificial intelligence and computer-aided diagnosis programs have emerged as new methods to provide more confidence in the imaging diagnosis. The use of machine learning algorithms in bladder medical image analysis can save time for the practitioners, can lead to improved performance for the radiologists and higher reporting accuracy. Such algorithms could be prerequisites for the next step of quantifying muscle invasiveness of the tumor with the aim of differentiating between e.g., non-muscle invasive and muscle-invasive bladder tumor (NMIBC vs MIBC). These algorithms can be integrated into an intelligent decision support system using methods that point beyond the existing state-of-the-art methods.

## 5 Limitations

Our study is based on a limited, but carefully selected number of clinical data from a single center, which were obtained on a single MRI scanner. Although the electronic database was prospective, the data analysis was performed retrospectively. Prospective and multicenter validation of our results is warranted.

In terms of weaknesses, the system requires pre-processed data to be trained, thus cannot be used as a prediction tool for raw MRIs, which hinders the overall process by this separated data processing step using the ground truth before introducing it into the system. A broader dataset would improve the model’s performance, which would improve the segmentation results.

## 6 Conclusions

We developed an intelligent system for automated identification of bladder tumors, that uses a deep learning model to segment both the bladder wall and the tumor only using 3D T2 MRI images. The U-Net based models showed promising results and are well-suited for the task of bladder segmentation. Even though all the tested models produce satisfactory outcomes, there are certain aspects that could be further optimized, as the future of decision support systems is to precisely determine the location of the tumor in relation to the organ wall and its layers.

## Data availability statement

The original contributions presented in the study are included in the article/**Supplementary Material**. Further inquiries can be directed to the corresponding authors.

## Ethics statement

The studies involving human participants were reviewed and approved by Ethical Commission of the Iuliu Hatieganu University of Medicine and Pharmacy, 400012 Cluj-Napoca, Romania. Written informed consent for participation was not required for this study in accordance with the national legislation and the institutional requirements.

## Author contributions

Conception and design, ZB, LD, IA, NC; study materials and patients, LD, AA, NC, PM, IA, CC, AL; formal data analysis, DC, LD, BB, TT; investigation, DC, NC, PM, IA, TT, CC, AL, BB; software development DC, LD, AA; data curation, IA, TT; data processing, DC, LD, TT, ZB, writing—original draft preparation, DC, LD, IA, ZB; writing—review and editing, DC, LD, NC, PM, IA, TT, AA, CC, AL, ZB, BB; final approval of manuscript, DC, LD, NC, PM, IA, TT, AA, CC, AL, BB, ZB; visualization, DC, LD; supervision, CC, AL, ZB; project administration, ZB; funding acquisition, NC.
