# Supplementary material for: Fully automated bladder tumor segmentation from T2 MRI images using 3D U-Net algorithm
Source: Front Oncol. 2023 Mar 9;13:1096136. doi: 10.3389/fonc.2023.1096136 (PMC10033524; doi:10.3389/fonc.2023.1096136)
Supplement: Supplementary file 1 [file DataSheet_1.pdf]

Supplementary Material

1.1 Supplementary Figures

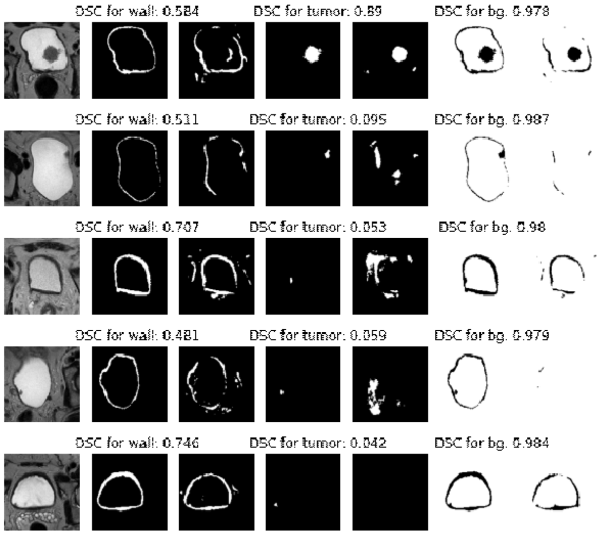

Supplementary Figure 1. Representative results on 5 validation datasets of the U-Net-4 algorithm using 10 augmentations. Each row present one image slice as example, followed by the manual annotation of the bladder wall (second column), the result of the algorithm on the bladder wall (third column), manual annotation of the tumor (forth column), the result of the algorithm on the tumor (fifth column), the background structures of the manual annotations (sixth column) and the result of the algorithm (seventh column).

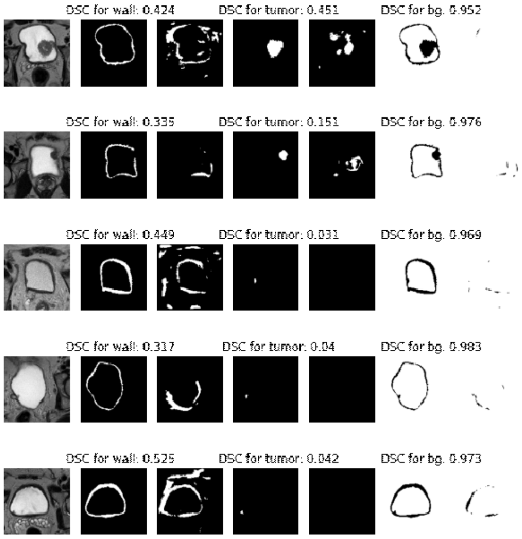

Supplementary Figure 2. Representative results on 5 validation datasets of the U-Net-4 algorithm using 5 augmentations. Each row present one image slice as example, followed by the manual annotation of the bladder wall (second column), the result of the algorithm on the bladder wall (third column), manual annotation of the tumor (forth column), the result of the algorithm on the tumor (fifth column), the background structures of the manual annotations (sixth column) and the result of the algorithm (seventh column).

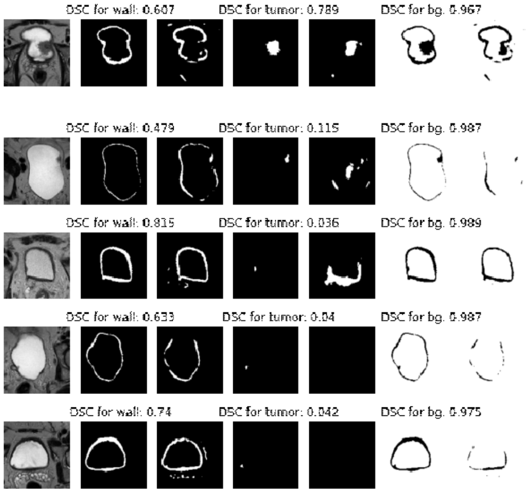

Supplementary Figure 3. Representative results on 5 validation datasets of the U-Net-5 algorithm using 10 augmentations. Each row present one image slice as example, followed by the manual annotation of the bladder wall (second column), the result of the algorithm on the bladder wall (third column), manual annotation of the tumor (forth column), the result of the algorithm on the tumor (fifth column), the background structures of the manual annotations (sixth column) and the result of the algorithm (seventh column).

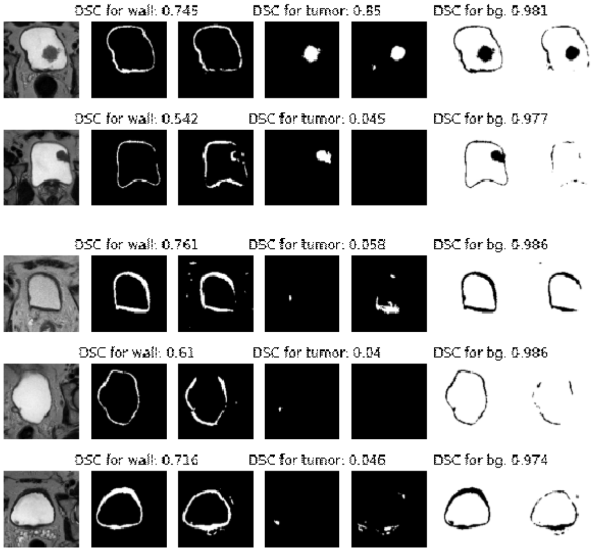

Supplementary Figure 4. Representative results on 5 validation datasets of the U-Net-6 algorithm using 10 augmentations. Each row present one image slice as example, followed by the manual annotation of the bladder wall (second column), the result of the algorithm on the bladder wall (third column), manual annotation of the tumor (forth column), the result of the algorithm on the tumor (fifth column), the background structures of the manual annotations (sixth column) and the result of the algorithm (seventh column).

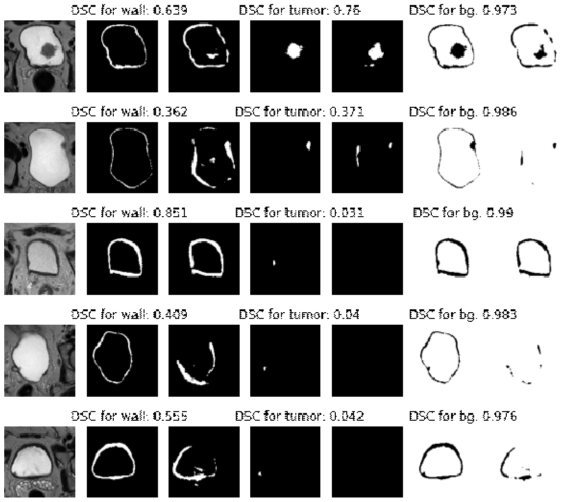

Supplementary Figure 5. Representative results on 5 validation datasets of the U-Net-6 algorithm using 5 augmentations. Each row present one image slice as example, followed by the manual annotation of the bladder wall (second column), the result of the algorithm on the bladder wall (third column), manual annotation of the tumor (forth column), the result of the algorithm on the tumor (fifth column), the background structures of the manual annotations (sixth column) and the result of the algorithm (seventh column).
